# Third national surveillance of risk factors of non-communicable diseases (SuRFNCD-2007) in Iran: methods and results on prevalence of diabetes, hypertension, obesity, central obesity, and dyslipidemia

**DOI:** 10.1186/1471-2458-9-167

**Published:** 2009-05-29

**Authors:** Alireza Esteghamati, Alipasha Meysamie, Omid Khalilzadeh, Armin Rashidi, Mehrdad Haghazali, Fereshteh Asgari, Mandana Kamgar, Mohammad Mehdi Gouya, Mehrshad Abbasi

**Affiliations:** 1 Endocrinology and Metabolism Research Center (EMRC), Vali-Asr Hospital, Tehran University of Medical Sciences, Tehran, Iran; 2 Department of Community Medicine, School of Medicine, Tehran University of Medical Sciences, Tehran, Iran; 3 Center for Disease Control, Ministry of Health and Medical Education, Tehran, Iran

## Abstract

**Background:**

The burden of non-communicable diseases is rising globally. This trend seems to be faster in developing countries of the Middle East. In this study, we presented the latest prevalence rates of a number of important non-communicable diseases and their risk factors in the Iranian population.

**Methods:**

The results of this study are extracted from the third national Surveillance of Risk Factors of Non-Communicable Diseases (SuRFNCD-2007), conducted in 2007. A total of 5,287 Iranian citizens, aged 15–64 years, were included in this survey. Interviewer-administered questionnaires were applied to collect the data of participants including the demographics, diet, physical activity, smoking, history of hypertension, and history of diabetes. Anthropometric characteristics were measured and serum biochemistry profiles were determined on venous blood samples. Diabetes (fasting plasma glucose ≥ 126 mg/dl), hypertension (systolic blood pressure ≥ 140 mmHg, diastolic blood pressure ≥ 90 mmHg, or use of anti-hypertensive drugs), dyslipidemia (hypertriglyceridemia: triglycerides ≥ 150 mg/dl, hypercholesterolemia: total cholesterol ≥ 200 mg/dl), obesity (body mass index ≥ 30 kg/m^2^), and central obesity (waist circumference ≥ 80 cm in females and ≥ 94 cm in males) were identified and the national prevalence rates were estimated.

**Results:**

The prevalence of diabetes, hypertension, obesity, and central obesity was 8.7% (95%CI = 7.4–10.2%), 26.6% (95%CI = 24.4–28.9%), 22.3% (95%CI = 20.2–24.5%), and 53.6% (95%CI = 50.4–56.8%), respectively. The prevalence of hypertriglyceridemia and hypercholesterolemia was 36.4% (95%CI = 34.1–38.9%) and 42.9% (95%CI = 40.4–45.4%), respectively. All of the mentioned prevalence rates were higher among females (except hypertriglyceridemia) and urban residents.

**Conclusion:**

We documented a strikingly high prevalence of a number of chronic non-communicable diseases and their risk factors among Iranian adults. Urgent preventive interventions should be implemented to combat the growing public health problems in Iran.

## Background

Chronic non-communicable diseases (NCDs) such as cardiovascular disease and diabetes are the leading cause of death worldwide [1], having comprised 60% of all deaths in 2005. Approximately 80% of NCD-attributable deaths are occurring in low and middle-income countries [2]. Furthermore, NCDs were responsible for nearly half of the burden (measured in disability-adjusted life years [DALYs]) of diseases in 2005, both worldwide and in low/middle-income countries [3]. The global prevalence of NCDs is increasing, with the majority of cases occurring in developing countries [4]. In this context, the Middle East is expected to bear one of the world's greatest increases in the absolute burden of NCDs and their risk factors in the near future. Most of this increase is anticipated to affect the economically productive age of 45 to 64 years, in contrast to most developed countries in which the increase in chronic disease burden concerns mainly the ages above 65 years [5-7]. The increasing burden of NCDs is especially prominent among urban dwellers, and is consistent with the epidemiological transition from communicable to non-communicable chronic diseases as the predominant causes of morbidity in developing populations [8,9]. The age-standardized death rate attributable to cardiovascular diseases and diabetes is estimated to be higher than 400 per 100,000 in Iran, one of the largest populations in the Middle East. The loss of the Iranian gross domestic product (GDP) due to heart disease and diabetes in 2015 will be 167% of that in 2006 [3].

The results of the Asia-Pacific Cohort Studies Collaboration (APCSC) project have improved our insight to the prevalence of NCDs and their consequences in the Asia-Pacific region [10]. Overweight and obesity are endemic problems in much of the region. The population-attributable fractions in this region because of overweight and obesity is up to 9.2% for coronary heart disease mortality, 2.9% for hemorrhagic stroke mortality, and 10.2% for ischemic stroke mortality [11]. According to the nationally representative data available from 12 countries in the region, diabetes has a prevalence of between 2.6% to 15.1%. The population-attributable fractions because of diabetes is up to 12% for coronary heart disease mortality, 6% for hemorrhagic stroke mortality, and 11% for ischemic stroke mortality [12]. The prevalence of hypertension in the region is up to 47% in men and 38% in women. The population-attributable fractions for coronary heart disease mortality, hemorrhagic stroke mortality and ischemic stroke mortality in men (women) are up to 39%, 66% (49%), and 44% (45%), respectively [13]. The prevalence of high serum total cholesterol (TC) in the region ranges from 4% to 27%. Up to 14% and 15%, respectively, of coronary artery disease mortality and ischemic disease mortality is attributable to high TC [14].

The results from Middle-Eastern countries are scant, mainly because of the rarity of nationally representative data in these countries. In order to determine the magnitude of the problem associated with NCDs in the population of Iran, the national surveys of NCDs risk factors have been conducted annually since 2005 under the supervision and recommendations of the World Health Organization (WHO). The first, second and the third surveys were performed in 2005, 2006 and 2007. The third national Surveillance of Risk Factors of Non-Communicable Diseases (SuRFNCD) provided the demographic, anthropometric and biochemical characteristics of a nationally representative sample of 5,278 Iranian adults aged 15–64 as well as valuable information on their diet and physical activity. In this study, we aimed to describe the methodology applied to conduct this survey and to present the national prevalence of a number of important non-communicable health problems and their risk factors including diabetes, hypertension, obesity, central obesity, and dyslipidemia.

## Methods

The third national SuRFNCD was conducted in March 2007 and comprised 5,287 non-institutionalized Iranian adults aged 15–64 years. Institutionalized individuals such as soldiers and those living in nursing homes were not included. After excluding participants aged 15 to 24 years (n = 1,054), analysis was performed on data of the remaining 4,233 individuals, who were aged 25–64 years. The reason for this exclusion was related to the relatively rapidly changing BMI of individuals between 15 and 24 years of age. The appropriate method to define obesity in this age group is by using percentiles in which case comparisons with adult obesity would not be trivial and may negatively affect the results.

The study was designed based on the STEPwise guidelines of the WHO [15]. The survey received ethics approval of the Center for Disease Control (CDC) of Iran and was carried out in collaboration with 40 medical schools across the country. All participants gave verbal informed consent. The participants were recruited in clusters of 10 males and 10 females living in neighboring households. The address of the first subject in each cluster was selected randomly out of the postal addresses and sampling was continued based on a predetermined schedule to register all 20 participants. The target population was adults aged 15 to 64 years, classified into 5 ten-year age groups (i.e. 15–24, 25–34, 35–44, 45–54, and 55–64). Each cluster comprised two males and two females in each age group. The number of clusters selected from each province was proportional to the urban/rural size of that province. For example, 51 clusters were taken from Tehran and only 2 from Ilam (the smallest of the 30 provinces of Iran). The participants were visited at their household by interviewers who were recommended by the collaborating medical schools to the managerial team in CDC. Interviewers were trained and instructed on the details of the survey in a one-day workshop in Tehran prior to the commencement of the survey. Informed consents were obtained and the required information was recorded in the following three steps. In step 1, general health characteristics and demographic information were collected by standardized questionnaires. In step 2, physical examination was performed to determine weight, height, waist circumference, and blood pressure. Participants were subsequently invited to prepare for step 3, i.e. collection of 10–12 hour fasting blood samples.

### Step 1: Demographic Data and Behavioral Assessments

Interviewer-administered questionnaires based on WHO STEPS instrument (core and expanded) were filled out in 6 different domains: demographic information, diet, physical activity, tobacco use, history of hypertension, and history of diabetes. Demographic information was comprised of insurance coverage and occupation in addition to sex, age, province of residence, residential area (urban/rural) and the postal address.

To identify participants with known diabetes mellitus (KDM), they were asked if a health care professional had ever told them that they had diabetes. History of diabetes in the first degree relatives was also recorded. For determination of access to care, participants were asked whether a health care professional has told them to have diabetes during the past 12 month, and whether they have had a blood glucose test during the past 12 months. Diabetic individuals were further asked whether their physician had recommended life style and nutrition modification, or had prescribed insulin and/or oral agents for them.

Regarding the history of hypertension, consumption of anti-hypertensive medication in the past 2 weeks, the time of the most recent blood pressure measurement by a health care professional, and whether a health care professional has ever told the participant to have hypertension were recorded.

### Step 2: Physical Examination

Weight and height of participants were determined in light clothing and without shoes. Portable calibrated electronic weighing scale and portable measuring inflexible bars were used. Waist circumference (WC) was measured using constant tension tape at the end of a normal expiration, with arms relaxed at the sides, at the midpoint between the lower part of the lowest rib and the highest point of the hip on the mid-axillary line. Blood pressure was measured with a calibrated Omron M7 sphygmomanometer (HEM-780-E). The average of three measurements, made at intervals of 5 minutes, was used for analysis.

### Step 3: Biochemical Measurements

10 ml of venous blood was taken in sitting position, collected in 4 tubes, centrifuged immediately, and transferred under cold chain condition to the Central Reference Laboratory of Ministry of Health of Iran (Tehran, Iran). Fasting plasma glucose (FPG), total cholesterol (TC), high density lipoprotein cholesterol (HDL-C), low density lipoprotein cholesterol (LDL-C) and triglycerides (TG) were measured. One tube was treated with 2 μg sodium fluoride for glucose preservation to enhance the accuracy of glucose measurement. FPG was measured by the enzymatic colorimetric method using glucose oxidize test (intra- and inter-assay coefficients of variation 2.1% and 2.6%, respectively). Serum TC, TG, LDL-C and HDL-C were determined by enzymatic methods (Parsazmun, Karaj, Iran). The two remaining tubes were transferred to the endocrine laboratory of Vali-Asr hospital (Tehran University of Medical sciences, Tehran, Iran) for insulin, C reactive protein, and leptin measurements, which are not the subjects of this report.

### Definition of variables

We designated participants as having known diabetes mellitus (KDM) if a health care professional had ever told them to have diabetes. In those without KDM, FPG = 126 mg/dl was regarded as newly diagnosed diabetes. Impaired fasting glucose (IFG) was defined in those without KDM by FPG levels ≥ 100 mg/dl (5.6 mmol/l) but < 126 mg/dl (7.0 mmol/l) [16]. Hypertension was defined as systolic blood pressure ≥ 140 mmHg, diastolic blood pressure ≥ 90 mmHg, or current use of anti-hypertensive drugs. Pre-hypertension was defined as 120 mm Hg ≥ systolic blood pressure < 140 mmHg or 80 mmHg ≤ diastolic blood pressure < 90 mmHg in non-hypertensive participants [17]. The body mass index (BMI; calculated as weight/height^2^) ≥ 30 kg/m^2 ^and 25 ≤ BMI < 30 kg/m^2 ^were regarded respectively as obesity and overweight [18]. Central obesity was defined by the International Diabetes Federation (IDF) criteria (WC ≥ 80 cm in females and ≥ 94 cm in males) [19] and the criteria set by the National Cholesterol Education Program-Third Adult Treatment Panel (ATP III) criteria (WC ≥ 88 cm in females and ≥ 102 cm in males) [20]. For high TC, the cut points of 200 mg/dl and 240 mg/dl were considered. High TG was defined as TG ≥ 150 mg/dl [19].

### Statistical analysis

Complex sample survey analysis was performed in SPSS 16 for Windows (Chicago, IL, USA). To extrapolate the results to the Iranian adult population, the data were weighted for age (10-year strata), sex, and residence area (rural/urban) according to the results of the national census of Iran in 2006 (n = 31,409,737, age: 25–64 years [21]). The complex sample analysis plan was defined based on the clusters of sampling protocol, strata (age groups, sex, and residential area) and the determined weights. National estimates, made in the complex survey analysis mode, are expressed as mean ± standard error of the mean (SEM) or prevalence (95%CI).

## Results

Since the questionnaires were filled out by interviewers, missing data were extremely rare. For biochemical measurements, however, 836 (19.7%) participants did not consent to blood sampling. Biochemical measurements were thus available for the remaining 3,397 individuals. As presented in Table 1, the total prevalence of diabetes was estimated to be 8.7% (95%CI = 7.4–10.2), about half (47.1%) of which can be attributed to newly diagnosed disease. The prevalence of diabetes was higher among older age groups, females, and urban dwellers. The prevalence of IFG was 9.2% among non-diabetic individuals.

**Table 1:** Estimates of prevalence of newly diagnosed diabetes, known diabetes and IFG among Iranian adults 25–64 years old

	**Impaired Fasting Glucose**^ab^		**Known DM**		**New DM**^a^		**New and Known DM**^a^	
	**National estimate**^c^	**Prevalence** **(95% CI)**	**National estimate**^c^	**Prevalence** **(95% CI)**	**National estimate**^c^	**Prevalence** **(95% CI)**	**National estimate**^c^	**Prevalence** **(95% CI)**

**Age**								
25–34 (n = 843)	0.6	5.1 (3.4–7.6)	0.1	0.7 (0.3–1.5)	0.3	2.5 (1.4–4.4)	0.4	3.2 (2.0–5.0)
35–44 (n = 902)	0.9	10.0 (7.6–13.0)	0.4	4.9 (3.3–7.3)	0.4	4.3 (3.2–5.8)	0.8	9.2 (7.0–12.1)
45–54 (n = 869)	0.9	13.6 (11.5–16.1)	0.5	8.4 (6.7–10.4)	0.4	5.6 (3.5–8.7)	0.9	14.0 (11.2–17.3)
55–64 (n = 783)	0.5	14.6 (11.9–17.9)	0.4	12.1 (8.2–17.4)	0.2	6.7 (4.9–9.1)	0.6	18.8 (14.7–23.6)
**Sex**								
Males (n = 1645)	1.5	9.3 (7.7–11.3)	0.7	4.4 (3.1–6.1)	0.6	4.0 (3.1–5.2)	1.3	8.4 (6.6–10.5)
Females (n = 1752)	1.4	9.0 (7.2–11.3)	0.8	4.9 (3.8–6.2)	0.7	4.2 (2.9–6.0)	1.4	9.1 (7.4–11.2)
**Residential area**								
Urban (n = 2175)	2.1	9.8 (8.3–11.4)	1.1	4.9 (3.9–6.2)	1.0	4.3 (3.4–5.4)	2.1	9.2 (7.8–10.9)
Rural (n = 1222)	0.7	7.8 (5.4–11.1)	0.3	3.9 (2.4–6.1)	0.3	3.7 (2.1–6.3)	0.7	7.5 (5.2–10.9)
**Total national estimate** (n = 3397)	2.9	9.2 (7.9–10.7)	1.5	4.6 (3.8–5.7)	1.3	4.1 (3.3–5.1)	2.7	8.7 (7.4–10.2)

DM: Diabetes mellitus, CI: confidence interval

^a ^In subjects with valid fasting plasma glucose measurements

^b ^In non-diabetic subjects

^c ^Rounded to the nearest million

Estimates are weighted for age, sex, and residential area on the basis of the population of Iran in 2006

The national estimate of BMI was 26.47 ± 0.15 kg/m^2^. The prevalence of obesity and overweight was 22.3% (95%CI = 20.2–24.5) and 36.3% (95%CI = 34.6–38.1), respectively. Obesity was more prevalent among females and urban dwellers (Table 2). The mean WC of Iranian adults was estimated to be 88.67 ± 0.38 cm. The prevalence of central obesity, defined by the ATP III and IDF criteria, were respectively 33.8% (54.4% in females and 13.9% in males) and 53.6% (73.4% in females and 34.4% in males) (Table 3). Irrespective of the criteria used, the prevalence of central obesity grew with increasing age, and was higher among females and urban residents.

**Table 2:** Estimates of prevalence of obesity among Iranian adults 25–64 years old

	**Obesity**		**Overweight**^a^	
	**National** **estimate**^b^	**Prevalence** **(95% CI)**	**National** **estimate**^b^	**Prevalence** **(95% CI)**

**Age**				
25–34 (n = 1081)	1.8	14.4 (11.5–17.8)	4.1	31.8 (29.0–34.7)
35–44 (n = 1113)	2.4	26.7 (22.7–31.1)	3.5	39.4 (36.1–42.8)
45–54 (n = 1069)	1.8	29.3 (25.6–33.2)	2.5	39.8 (36.4–43.3)
55–64 (n = 970)	0.9	27.4 (24.1–31.0)	1.3	39.3 (35.1–43.6)
**Sex**				
Males (n = 2121)	2.3	14.2 (12.2–16.5)	6.0	37.5 (35.0–40.1)
Females (n = 2112)	4.7	30.6 (27.3–34.0)	5.4	35.1 (32.8–37.6)
**Residential area**				
Urban (n = 2853)	5.3	23.8 (21.5–26.2)	8.6	38.2 (36.2–40.2)
Rural (n = 1380)	1.7	18.5 (14.5–23.4)	2.8	31.7 (28.4–35.3)
**Total national estimate** (n = 4233)	7.0	22.3 (20.2–24.5)	11.4	36.3 (34.6–38.1)

CI: confidence interval

^a ^In non-obese subjects

^b ^Rounded to the nearest million

Estimates are weighted for age, sex, and residential area on the basis of the population of Iran in 2006

**Table 3:** Estimates of prevalence of central obesity defined by the ATP III and the IDF criteria among iranian adults 25–64 years old

	**High WC by IDF**^a^		**High WC by ATPIII**	
	**National** **estimate**^b^	**Prevalence** **(95% CI)**	**National** **estimate**^b^	**Prevalence** **(95% CI)**

**Age**				
25–34 (n = 1081)	5.0	39.3 (34.2–44.6)	2.7	21.5 (17.5–26.1)
35–44 (n = 1113)	5.3	58.7 (53.7–63.5)	3.3	37.5 (31.8–43.5)
45–54 (n = 1069)	4.2	66.7 (61.2–71.8)	2.9	45.5 (39.0–52.2)
55–64 (n = 970)	2.3	70.0 (64.7–74.7)	1.7	49.5 (42.6–56.5)
**Sex**				
Males (n = 2121)	5.5	34.4 (31.3–37.5)	2.2	13.9 (11.9–16.0)
Females (n = 2112)	1.1	73.4 (69.5–77.0)	8.4	54.4 (50.0–58.8)
**Residential area**				
Urban (n = 2853)	12.6	56.0 (52.5–59.5)	8.0	35.5 (32.0–39.1)
Rural (n = 1380)	4.2	47.5 (40.7–54.4)	2.7	29.8 (23.8–36.5)
**Total national estimate** (n = 4233)	16.8	53.6 (50.4–56.8)	10.6	33.8 (30.8–37.1)

WC: waist circumference, CI: confidence interval

^a ^ATP III: Third Adult Treatment Panel; IDF: International Diabetes Federation

^b ^Rounded to the nearest million

Estimates are weighted for age, sex, and residential area on the basis of the population of Iran in 2006

The estimated systolic and diastolic blood pressure was 122.84 ± 0.57 mmHg and 80.67 ± 0.39 mmHg, respectively. Overall 26.6% (95%CI = 24.4–28.9) of Iranian adults were estimated to be hypertensive and an additional 38.2% (95%CI = 36.1–40.2; corresponding to 12 million people) to have prehypertension. Hypertension was more prevalent among older age groups, females and urban residents (Table 4).

**Table 4:** Estimates of prevalence of hypertension and pre-hypertension among iranian adults 25–64 years old

	**Pre-hypertension**^a^		**Hypertension**	
	**National** **estimate**^b^	**Prevalence** **(95% CI)**	**National** **estimate**^b^	**Prevalence ** **(95% CI)**

**Age**				
25–34 (n = 1081)	5.0	39.0 (35.3–42.8)	1.8	14.0 (11.5–17.0)
35–44 (n = 1113)	3.6	39.8 (36.1–43.7)	2.3	25.2 (22.6–28.1)
45–54 (n = 1069)	2.5	39.1 (35.9–42.4)	2.4	38.4 (35.0–41.9)
55–64 (n = 970)	1.0	28.7 (25.5–32.0)	1.9	56.4 (52.4–60.3)
**Sex**				
Males (n = 2121)	7.1	44.3 (41.3–47.3)	3.9	24.7 (22.1–27.4)
Females (n = 2112)	4.9	31.9 (29.4–34.4)	4.4	28.6 (25.1–32.3)
**Residential area**				
Urban (n = 2853)	8.6	38.0 (35.7–40.4)	6.3	28.2 (25.7–30.9)
Rural (n = 1380)	3.4	38.4 (34.4–42.6)	2.0	22.6 (18.6–27.1)
**Total national estimate** (n = 4233)	12.0	38.2 (36.1–40.2)	8.4	26.6 (24.4–28.9)

CI: confidence interval

^a ^In non-hypertension subjects

^b ^Rounded to the nearest million

Estimates are weighted for age, sex, and residential area on the basis of the population of Iran in 2006

The national estimates of TG and TC were 148.83 ± 2.45 mg/dl and 195.63 ± 1.10 mg/dl, respectively. The prevalence of hypertriglyceridemia, TC ≥ 200 mg/dl, and TC ≥ 240 mg/dl were 36.4% (95%CI = 34.1–38.9), 42.9% (95%CI = 40.4–45.4), and 14.1% (95%CI = 12.6–15.9), respectively. Dyslipidemia was more common among urban dwellers and older age groups. Females had a higher prevalence of hypercholesterolemia while males had a higher prevalence of hypertriglyceridemia (Table 5).

**Table 5:** Estimates of prevalence of high triglycerides levels and hypercholesterolemia among Iranian adults 25–64 years old

	**Triglycerides ≥ 150 mg/dl**		**Cholesterol ≥ 200 mg/dl**		**Cholesterol ≥ 240 mg/dl**	
	**National ** **estimate**^a^	**Prevalence** **(95% CI)**	**National ** **estimate**^a^	**Prevalence** **(95% CI)**	**National ** **estimate**^a^	**Prevalence** **(95% CI)**

**Age**						
25–34 (n = 843)	3.5	27.1 (23.7–30.7)	3.9	30.2 (26.8–33.7)	1.1	8.8 (6.6–11.6)
35–44 (n = 902)	3.8	42.4 (37.4–47.7)	4.2	46.2 (42.5–50.0)	1.3	14.2 (11.4–17.6)
45–54 (n = 869)	2.7	42.6 (39.3–45.9)	3.4	54.0 (50.3–57.7)	1.2	19.0 (16.6–21.6)
55–64 (n = 783)	1.5	44.5 (40.7–48.4)	2.1	61.6 (56.3–66.6)	0.8	25.4 (21.7–29.5)
**Sex**						
Males (n = 1645)	6.3	39.6 (36.0–43.3)	6.4	40.4 (36.8–44.2)	1.8	11.0 (9.0–13.4)
Females (n = 1752)	5.1	33.2 (30.3–36.2)	7.0	45.4 (42.1–48.7)	2.7	17.3 (15.1–19.8)
**Residential area**						
Urban (n = 2175)	8.8	39.3 (36.4–42.2)	9.9	44.2 (41.4–47.0)	3.2	14.4 (12.7–16.4)
Rural (n = 1222)	2.6	29.3 (26.1–32.8)	3.5	39.6 (34.7–44.6)	1.2	13.4 (10.3–17.3)
**Total national estimate** (n = 3397)	11.4	36.4 (34.1–38.9)	13.5	42.9 (40.4–45.4)	4.4	14.1 (12.6–15.9)

CI: confidence interval

^a ^Rounded to the nearest million

Estimates are weighted for age, sex, and residential area on the basis of the population of Iran in 2006 Iran

## Discussion

The WHO STEPS surveillance program was designed to generate validated and internationally comparable data about the chronic non-communicable or cardiovascular diseases particularly for populations with less available evidences. Our third national SuRFNCD was conducted in 2007 based on the WHO STEPS guidelines. The main results of this survey indicate the high prevalence of diabetes, hypertension, obesity, central obesity, and dyslipidemia in Iran. Overall these problems were more common among females, older age groups, and in urban areas. In a large study (age: 15–64 years) in the North-East of Iran, sociodemographic factors such as progressive urbanization and advancing age were significantly correlated to the increasing prevalence of type 2 diabetes [22]. Similarly, the prevalence of diabetes was linked to urbanization in a recent study (age > 19 years) in central Iran [23]. Hypertension was significantly more common among females than males in another large study (age > 19 years) in central Iran [24]. Speedy urbanization and advancing age are not the only reasons underlying the increasing prevalence of NCDs in Iran. Nutritionally-related health patterns have changed dramatically in the Middle-East during recent years, partly because of social development in the absence of steady economic growth. Changes in dietary and physical activity patterns as well as inequality in health care are other important factors [25]. In the following sections, our findings are discussed and compared to the reports from the United States, western European countries, our Asian neighbors, and other Asian countries.

### Diabetes

**Figure 1:**
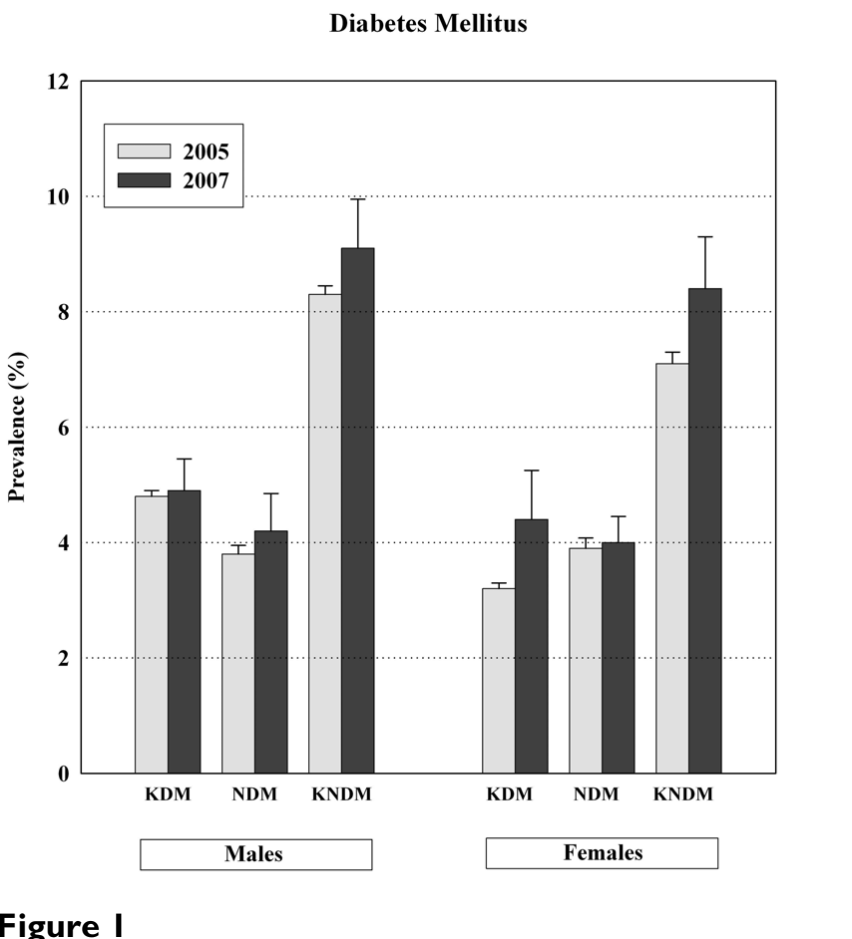
**Comparison between 2005 and 2007 in prevalence of diabetes among Iranian adults**. KDM: known diabetes mellitus, NDM: newly diagnosed diabetes mellitus, KNDM: known and new diabetes mellitus.

The APCSC study reports prevalence rates between 2.6% and 15.1% for countries in the Asia-Pacific region [12]. We have previously reported the national prevalence of diabetes as 7.7% (8.3% in females and 7.1% in males, age: 25–64 years) in 2005 [26]. According to the results of the present study, the prevalence of diabetes is about 8.7% in Iranians aged 25–64 years old (9.2% in females and 7.5% in males). A comparison between years 2005 and 2007 can be found in Figure 1. These estimates are a just below the reported rates from the United States (9.3%; 8.2% in females and 10.6% in males, age > 20 years) [27], but considerably higher than the estimates made in the UK (3.4%; age: 30–59 years) [28] and comparable to reports from Australia (7.4%; 6.8% in females and 8.0% in males, age range ≥ 25 years) [29]. The prevalence of diabetes in one of our neighbors, Turkey (11% in both females and males, age ≥ 35 years) [30], is similar to our prevalence rates (12.5%; 13.0% in females and 12.1% in males) in the same age range. Our prevalence rates are higher than those reported from China (5.5%; 5.8% in females and 5.2% in males, age: 35–74 years) [31] and comparable to the rates reported from Korea (7.6%; 7.5% in females and 8.1% in males, age ≥ 20 years) [32]. A higher prevalence is reported in India (12.1%, age ≥ 20 years) [33]. The rising prevalence of diabetes with increasing age in our study is consistent with the mentioned reports. The total prevalence of diabetes estimated in our study corresponds to more than 2.7 million adults, about half of whom are newly diagnosed cases. We further estimated that approximately 2.9 million non-diabetic Iranian adults suffer from IFG.

### Obesity

The APCSC study reports prevalence rates ranging from less than1% to higher than 20% for countries in the Asia-Pacific region [11]. Our study demonstrated the prevalence of obesity to be 22.3% among Iranian adults (30.6% in females and 14.2% in males), corresponding to about 7 million individuals. A review in 2005 estimated the prevalence of obesity among urban Iranians aged 15–70 years to be between 22% and 40% [34]. Another survey (2004; age: 20–70 years) in the north of Iran reached an estimate of 27.8% in females and 9.9% in males [35]. According to National Health and Nutrition Examination Survey (NHANES) of the US, the prevalence of obesity in individuals aged 20–74 years was 34% in females and 31.7% in males [36]. The corresponding figures in Australia (age ≥ 25 years) were 19% and 17%, respectively [37]. In the UK, the prevalence of obesity was estimated to be 24.2% in females and 23.7% in males (national Health Survey, 2006) [38]. Compared to the results from the US and UK, the prevalence of obesity among Iranian males is much lower. The higher prevalence of obesity in Iranian females, compared to males, is in agreement with the results from most of our neighboring Arab countries, including Saudi Arabia (24% in females and 16% in males, age ≥ 15 years) [39], Oman (23.8% in females and 16.7% in males, age ≥ 20 years) [40] and Lebanon (18.8% in females and 14.3% in males, age ≥ 20 years) [41]. In Turkey as well, the prevalence of obesity is higher in females (24.6% vs. 14.4% in males, age ≥ 20 years) [42]. Taken together, the prevalence of obesity among Iranian females exceeds the rates in females from our neighboring countries.

### Central Obesity

Approximately, 33.8% (54.4% in females and 13.9% in males, corresponding to more than 10.6 million adults) of Iranian adult population are centrally obese according to ATP III definition. A unique finding of our study is the strikingly higher prevalence of central obesity among females (about 4 times more than males) compared to males. A similar ratio (46.2% in females and 10.6% in males, age: 20–70 years) was obtained in 2004 in a survey in the north of Iran [35]. The prevalence of ATP III-defined central obesity was about 38.6% (46.3% in females and 29.8% in males) in the US [43] and 36.5% (41% in females and 32% in males) in the UK [38]. In comparison, central obesity is more common in Iranian females, and considerably less common in males. The prevalence of central obesity among females in Turkey (57.6%), one of our neighbors, is comparable to our estimate. The total prevalence of central obesity (40.9%) and the prevalence in males (21.2%) in Turkey (age ≥ 20 years) are higher than our estimates for Iran [44]. The prevalence among Tunisian males (8.8%) is lower than our estimate (age ≥ 20 years) [45]. Finally, central obesity is significantly less common in the Eastern Asian countries such as China [46] and Korea [47] than in Iran.

### Hypertension

**Figure 2:**
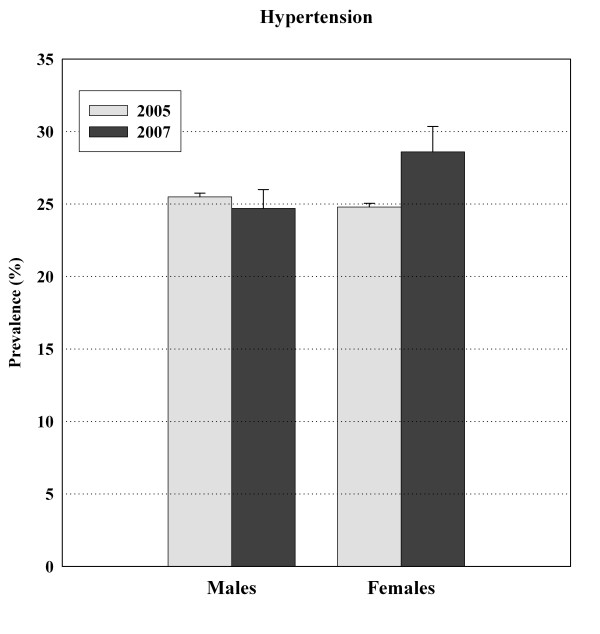
**Comparison between 2005 and 2007 in prevalence of hypertension among Iranian adults**.

The APCSC study reports prevalence rates between 5% and 47% in men and between 7% and 38% in women for countries in the Asia-Pacific region [13]. In our survey the prevalence rate of hypertension was 26.6% (28.6% in females and 24.7% in males), corresponding to 8.3 million adults. Our previous national estimate (2005) for the prevalence of hypertension was 25.2% (24.8% in females and 25.5% in males) [48]. A comparison between years 2005 and 2007 is provided in Figure 2. A systematic review in 2004 showed that the prevalence of hypertension varies greatly around the world, lowest in rural India (6.8% in females and 3.4% in males) and highest in Poland (72.5% in females and 68.9% in males) [49]. In Turkey, the prevalence is 31.8% (36.1% in females and 27.5% in males, age ≥ 18 years) [50], which is higher than our estimate. The prevalence in the 35–64 year old population of the US (27.8%; 25.8% in females and 29.8% in males) was lower than our estimate in a similar age range (35.3%; 40.1% in females and 30.7% in males), whereas reports from the UK (41.7%; 36.5% in females and 46.9% in males), Germany (55.3%; 50.3% in females and 60.2% in males) and Spain (46.8%; 44.6% in females and 49.0% in males) point to prevalence rates higher than our estimate [51]. The prevalence of hypertension in China in 2000–2001 (27.2%; 25.8% in females and 28.6% in males, age: 35–74 years) [52] was similar to our current estimate. According to the mentioned studies, hypertension grows in prevalence with increasing age.

### Hypertriglyceridemia

Data from previous reports on the prevalence of hypertriglyceridemia in Iran is rare. According to the latest results of the ongoing Tehran Lipid and Glucose Study (TLGS), 37.6% of Tehranian adolescents (age: 10–19 years) have TG levels above 110 mg/dl, defined as hypertriglyceridemia for their age range [53]. We estimated the prevalence of hypertriglyceridemia to be 36.4% among Iranian adults (33.2% in females and 39.6% in males). The figure is 30% in the US (age ≥ 20 years) [43], 25% in Sweden (age: 45–69 years) [54], 19.2% in Italy (age ≥ 20 years) [55], 12.5% in Switzerland (age: 35–75 years) [56] and approximately 18% in Portugal (age: 30–70 years) [57]. Our estimate is higher than the mentioned western countries. Similar reports from Saudi Arabia (40.3%; 33.7% in females and 47.6% in males) [58], Turkey (30.7%; 26.1% in females and 36.2% in males) [44] and Lebanon (35.3%; 22.6% in females and 52.4% in males) [59] suggest that hypertriglyceridemia is a more common problem in our region than in western countries.

### Hypercholesterolemia

Data from previous reports on the prevalence of hypercholesterolemia in Iran is rare. According to the recent results of TLGS, the mean level of total cholesterol among Tehranian adult females is approximately 200 mg/dl, suggesting that about half of the female population of Tehran suffer from hypercholesterolemia [60]. In our survey, the prevalence of total hypercholesterolemia (TC ≥ 200 mg/dl) was estimated to be 42.9% (45.4% in females and 40.4% in males). In 2005, The American Heart Association (AHA) reported the prevalence of hypercholesterolemia to be 48.2% (48.6% in females and 47.8% in males) in non-Hispanic white Americans aged ≥ 20 [61]. In UK, approximately 48% (48% in females and 48% in males) of adults aged 19–64 years had hypercholesterolemia [62]. The prevalence rates in these two developed countries, and in a rather similar age range to our study, are approximately only five percent higher than our estimate in a developing country. The prevalence rate in Portugal (56.7%; age: 30–70 years) [57] and Saudi Arabia (54%; age: 30–70 years) [58] is also about 10–15% higher than our prevalence rate. The difference between the latter two studies and ours of about 5 years in the age range may explain the difference in prevalence rates. Hypercholesterolemia was present in 32.8% (34.2% in females and 31.6% in males) of the Chinese aged 35–74 years [63], which is much lower than our estimates and most of the above countries. The rising trend of hypercholesterolemia with increasing age was observed in all of the above studies.

## Conclusion

We documented a strikingly high prevalence of a number of chronic non-communicable diseases and their risk factors in Iran, and showed that the prevalence of these metabolic abnormalities in our country, as a developing country in the nutritional and life style transition phase is comparable, if not higher, to most developed countries. With continuation and acceleration of urbanization, the prevalence of these disease conditions will likely escalate. Therefore, urgent preventive interventions on a national scale should target these highly prevalent metabolic abnormalities (e.g. diabetes, obesity, hypertension, and dyslipidemia). Based on our results, women and urban residents need to be the focus of more intensive attention. The main limitation with our study was the possibility of recall bias associated with questions regarding past medical history (e.g. diabetes, hypertension). The other limitation of our results stems from the fact that approximately one fifth of our participants did not consent to blood sampling. Since only the demographic and anthropometric characteristics of these individuals were analyzed, additional sources of bias might have affected our results. Finally, caution needs to be practiced in inter-country comparisons which are subject to misinterpretations due to differences in age groups, study design, sampling method, and the year in which each survey was carried out.

## Competing interests

The authors declare that they have no competing interests.

## Authors' contributions

AE provided clinical expertise throughout the project. AM supervised the analysis and manuscript preparation. OK performed the statistical analysis and drafted the manuscript. AR contributed to the literature review, and helped with the revized manuscript. MH participated in the design and coordination of the survey. FA and MMG conducted the survey. MK cooperated in drafting the manuscript. MA designed the manuscript structure and contributed to the writing. All authors read and approved the final manuscript draft.

## Pre-publication history

The pre-publication history for this paper can be accessed here:


